# High school students’ use of JUUL pod flavors before and after JUUL implemented voluntary sales restrictions on certain flavors in 2018

**DOI:** 10.1371/journal.pone.0243368

**Published:** 2020-12-15

**Authors:** Meghan E. Morean, Krysten W. Bold, Grace Kong, Deepa R. Camenga, Asti Jackson, Patricia Simon, Danielle R. Davis, Suchitra Krishnan-Sarin

**Affiliations:** 1 Department of Psychiatry, Yale School of Medicine, New Haven, Connecticut, United States of America; 2 Departments of Emergency Medicine and Pediatrics, Yale School of Medicine, New Haven, Connecticut, United States of America; University of California San Diego School of Medicine, UNITED STATES

## Abstract

**Objectives:**

In November 2018, JUUL, Inc. restricted sales of mango, fruit medley, crème brûlée, and cucumber pods to its website. Physical/online sales of tobacco, menthol, and mint pods continued. We examined rates of adolescent JUUL device and flavored pod use before and after restrictions were implemented to examine their potential impact.

**Methods:**

Students from 4 Connecticut high schools completed cross-sectional surveys in 2018 (N = 3170) and 2019 (N = 3074).

**Results:**

Compared to 2018, current (past-month) JUUL use decreased in 2019 (30.2–25.6%). Among current JUUL users, restricted flavor use (mango [62.8–36.9%]; cucumber [27.7–11.9%]; fruit [23.5–11.4%]; crème brûlée [12.3–5.0%]) and the total number of flavors used (2.09[SD = 1.71]-1.51[SD = 1.33]) decreased (p-values < .001), while mint pod use increased (62.0–68.6%, p < .01). Tobacco and menthol pod use remained stable.

**Conclusions:**

Following voluntary sales restrictions implemented by JUUL, Inc., restricted flavor use and the total number of flavors used decreased among JUUL users while mint use increased. Results suggest flavor restrictions may impact adolescents’ e-cigarette use. While 2020 Food and Drug Administration restrictions on mint pods may further limit youth JUUL use, research is needed to determine where youth acquire restricted flavors and if restrictions prompt increased popularity of unrestricted devices/e-liquids.

## Introduction

Electronic cigarettes (e-cigarettes) have been the most popular tobacco product used by youth in the U.S. since 2014; in 2019 27.5% of high school students reported e-cigarette use in the past month [1]. This increased prevalence partially has been driven by the availability of pod-based devices including JUUL [2, 3], which has captured the majority of the U.S. e-cigarette market share [4] and is especially popular among underage youth [3, 5].

Research indicates that JUUL appeals to youth due to its compact and sleek design, concealability, appealing flavors, and the ability to provide a “buzz” (presumably due to JUUL’s high nicotine content) [2, 5]. Perhaps in response to the mounting government and public criticism, JUUL uniquely enacted voluntary measures in November 2018 to restrict youth access to their products. Specifically, JUUL voluntarily restricted sales of mango, fruit medley, crème brûlée, and cucumber pods exclusively to its own website, which employed age verification technology designed to limit youth access [6]. Classic tobacco, Virginia tobacco, menthol, and mint flavor JUUL pods continued to be sold in brick-and-mortar stores and online through JUUL’s website and other websites. Prior to JUUL’s sales restrictions, all JUUL pods were available for purchase in brick-and-mortar stores and online, with mango and mint being the most preferred flavors among high school youth [7]. Based on prior research suggesting that tobacco and menthol flavors generally do not appeal strongly to youth [7], there was only modest concern about sales of these flavors continuing in brick-and-mortar retailers. However, given the established popularity of mint among youth [7], concerns were raised that mint JUUL pod use would increase further following the sales restrictions because mint JUUL pods remained available in physical stores. There also was reason to be concerned that former users of JUUL’s sweet/fruity flavors may switch to another brand that had not imposed a flavor ban.

Addressing this issue on a large scale, an analysis of e-cigarette sales data found that, following JUUL’s 2018 sales restrictions on mango, fruit medley, crème brûlée, and cucumber pods, an increase in the sale of fruit flavors by non-JUUL brands was observed in 2019, suggesting that brand switching was occurring to obtain desirable flavors [8]. In addition, overall sales of mint/menthol flavors increased dramatically by 2019 and were accompanied by modest increases in tobacco flavor sales [8]; it was estimated that JUUL captured 100% of the growth in mint/menthol sales and 91% of the growth in tobacco e-liquid sales [8]. While the Nielsen scanner data used in aforementioned study [8] could not differentiate legal sales made to adults from illegal sales made to youth, data from the 2019 National Youth Tobacco Survey (NYTS) suggested that rates of youth use of menthol/mint flavors may track with the national sales data, as the popularity of mint/menthol flavors increased in 2019 [1]. However, several factors limit the conclusions that could be drawn from the NYTS study. First, mint and menthol were combined into a single category, making it impossible to determine if mint, menthol, or the combination of the two flavors drove increased use in the mint/menthol category. It is important to differentiate these flavors based on prior research suggesting mint is popular among adolescents while menthol is not [7]. Second, questions about flavor use were not product-specific, making it impossible to evaluate how JUUL’s sales restrictions impacted adolescents’ JUUL pod use. Finally, data on flavor use were limited to youth who solely used e-cigarettes, excluding those who used other tobacco products; 2019 NYTS data suggest that 45.5% of adolescent e-cigarette users use other tobacco products [9].

The current study was designed to examine whether JUUL’s 2018 voluntary sales policy, which restricted cucumber, mango, crème brûlée, and fruit JUUL pod sales to its website while allowing mint, tobacco, and menthol flavors to be sold in physical stores and through online retailers, impacted 2019 rates of JUUL device use among high school students and/or flavored JUUL pod use among current JUUL users. Baseline data collection (2018) concluded approximately one month prior to the implementation of JUUL’s flavor restrictions, and 2019 data collection commenced approximately five months after the restrictions were in place. We hypothesized that, among past-month JUUL users, use of restricted pod flavors would decrease as would the total number of JUUL pod flavors used in the past month. Likely due to preferred flavors becoming difficult to obtain, we anticipated that the overall rate of JUUL device use also would decrease. Given the established popularity of mint pods among youth [7], and the continued availability of mint JUUL pods in physical stores, we expected to observe increased mint pod use among current JUUL users. Rates of tobacco and menthol JUUL pod use were expected to remain stable given their prior lack of popularity among youth [7].

## Materials and methods

Study procedures were approved by the Yale University Institutional Review Board (#1207010580), the school boards, and the participating schools. All procedures met the ethical standards included in the Helsinki Declaration of 1975 (revised in 2000). Passive parental permission was obtained prior to survey administration. Students were informed that participation was voluntary, and completing the survey indicated consent/assent. A waiver of written consent/assent was granted to preserve the anonymous nature of the study.

### Participants

In 2018 and 2019, four public high schools representing a convenience sample from New Haven and Fairfield Counties in Connecticut were invited to participate in our annual youth tobacco survey. The schools were chosen for inclusion based on an established relationship between the research team and the schools (e.g., schools had completed the survey in years prior) and that fact that these schools were drawn from different District Reference Groups (DRGs) in CT. DRGs are used to group similar schools based on several factors (e.g., family income, parental education/occupation, use of a language other than English at home). There are a total of 9 DRGs in CT, which can be divided into thirds representing high, middle, low. One of our schools represented the top tercile (high), two the second (middle), and one the third (low), suggesting that the included schools were reasonably representative of public schools in CT.

From May-October of 2018, all 3,730 students who attended school on the day(s) of survey administration were invited to complete an anonymous, in-school, computerized survey. In total, 3,170 students completed the survey (response rate 85%; 52.4% female, 60.4% Non-Hispanic White, 15.87[SD = 1.29] years old; 34.0% past-month use of any vaping device; 30.2% past-month JUUL use). In Spring (April-June) of 2019, 3,967 students from the same high schools were invited to complete the next iteration of the annual survey. In total, 3,075 completed the survey (response rate = 77.5%; 51.6% female; 55.2% non-Hispanic White, 15.99 [SD = 1.28] years old; 29.7% past-month use of any vaping device; 25.6% past-month JUUL use). Cross-sectional data from each wave were used for analyses.

### Procedures

With regard to survey development, our team has been conducting annual school surveys on youth tobacco use since 2013. Over time, the development of our annual surveys has been informed by focus groups with youth and yearly subject matter expert input (range 8–12 experts per year). They key questions analyzed in the current study (described below) had been included in the annual survey since 2015. In addition, the formatting of the questions, including the addition of pictures to help youth better identify products, is consistent with recommended best practices [10] and mirrors procedures implemented in nationally representative [11] and regional surveys [12].

### Measures

All measures were self-report (see S1 Survey for question text and response options for variables included in this study).

After viewing a picture of a JUUL/JUUL pods and reading a brief description of the device, participants reported on lifetime JUUL use (no/yes). Participants who indicated lifetime use then reported on frequency of use in the past 30 days. Individuals who endorsed using JUUL at least once in the past month were coded as past-month JUUL users. Individuals who indicated past-month JUUL use subsequently reported whether they had used each of the eight JUUL pod flavors during the past 30 days (i.e., classic tobacco, Virginia tobacco, menthol, mint, mango, fruit medley, cucumber, crème brûlée; each coded as no/yes) or had “not used any of these JUUL pods flavors in the past month” (which was an exclusive answer). A variable was created reflecting the total number of JUUL pod flavors used in the past month, with zero reflecting individuals who endorsed not using any of the JUUL pod flavors or who indicated “no” to using each individual flavor.

### Analytic plan

Given that the 2018 data were collected over two semesters (Spring/Fall), we first ran a series of chi-square analyses to evaluate whether significant differences emerged by semester for past-month JUUL use (total sample) or JUUL pod flavors used (past-month JUUL users), and an independent-samples t-test for the total number of flavors used (past-month JUUL users). No significant differences emerged, so we combined the data from Spring and Fall 2018 to represent overall use in 2018 to simplify the presentation of results.

Cochran-Mantel-Haenszel tests were used to assess changes in JUUL device use (within the total sample) and flavored pod use (among past-month JUUL users) from 2018 to 2019 that occurred concurrently within the four different schools. Additional confounding variables were not considered. The test yields two important results: 1) tests of homogeneity of the odds ratio (indicating, in this case, whether the relationships between year and JUUL device/pod use differed by school) and 2) tests of conditional independence (indicating whether the magnitude of the odds ratio for the main effect of year was statistically significant). An independent samples t-test was run to examine differences in the total number of JUUL pod flavors used in the past 30 days, which was followed up by a chi-square analysis to better understand any observed changes. Finally, to get a better sense of whether the total number of flavors used varied as a function of frequency of JUUL use, we ran separate crosstabs for 2018 and 2019 on the number of flavors used in the past month (simplified to 0 to 5 or more) by the following JUUL use categories that were derived from frequency of past-month JUUL use: once a week or less, 2–3 times per week, 4–6 times per week, and daily. Analyses excluded participants with missing data (JUUL device use 2018 [1/3170], 2019 [7/3075]; JUUL pod use 2018 [92/956], 2019 [74/787]). The majority of data that was treated as missing for JUUL pod use (2018 [82/92]; 2019 [62/74]) resulted from participants indicating that they did not know what JUUL pod flavor(s) they had tried in a previous question assessing lifetime use.

## Results

**Fig 1:**
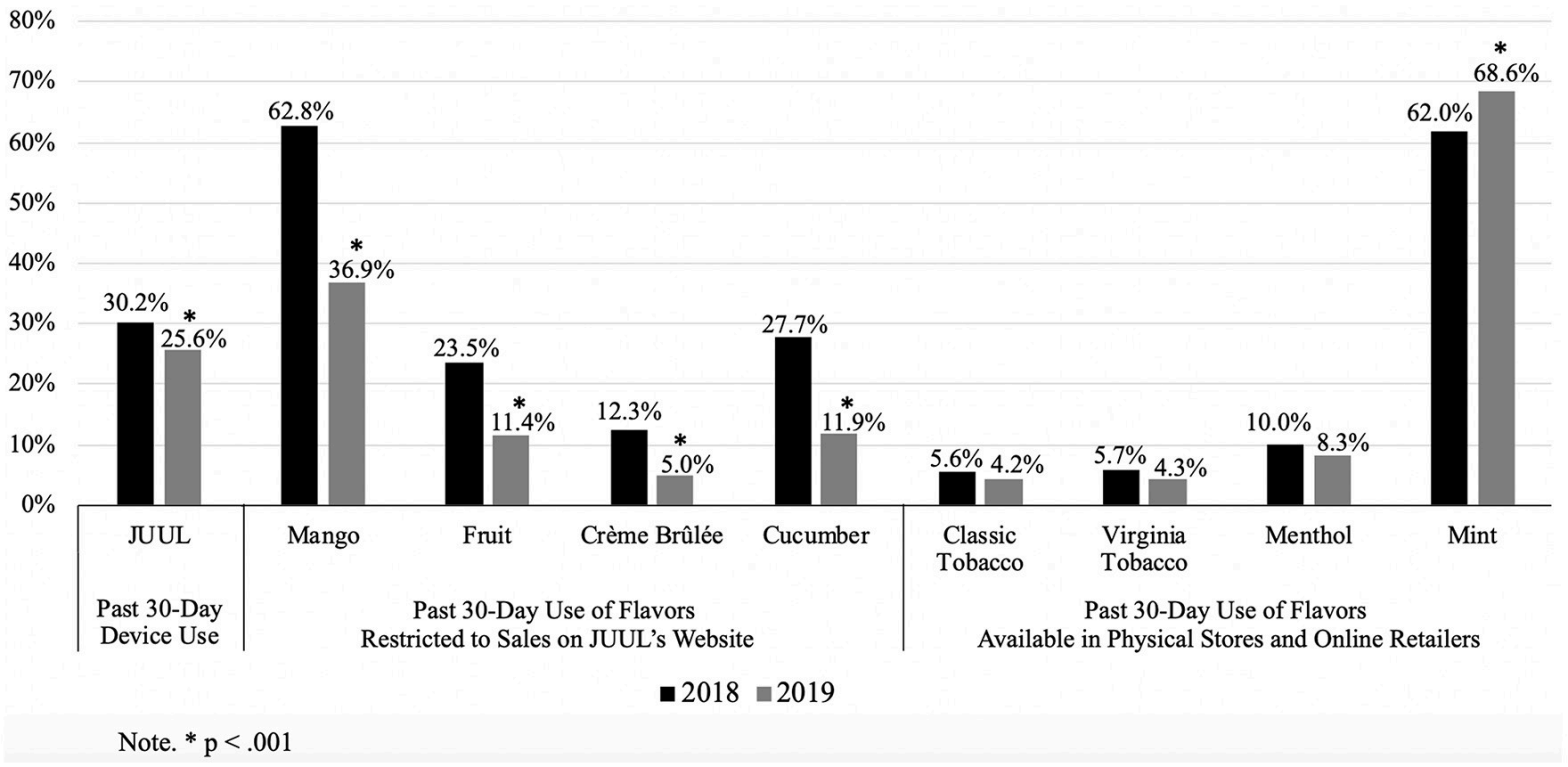
Rates of past 30-day use of JUUL and JUUL pod flavors in 2018 and 2019. *Note*. *p* < .001.

Across the Cochran-Mantel-Haenszel tests, no test of homogeneity of the odds ratio was significant, meaning that the relationships between JUUL device/pod use and year were not significantly different based on school (*p*-values ranged from 0.186 to 0.963). Results indicated that the rate of past 30-day JUUL device use decreased significantly from 2018 to 2019 among high school youth (30.2% vs. 25.6%, OR = 0.80 [0.72, 0.89], p < .001). Among past 30-day JUUL users, we observed decreases in rates of use of all JUUL pod flavors that were sold exclusively on JUUL’s website at the time of the 2019 survey (i.e., mango [62.8% vs. 36.9%, OR = 0.34 (0.28, 0.42)], fruit medley [23.5% vs. 11.4%, OR = 0.41 (0.31, 0.55)], crème brûlée [12.3% vs 5.0%, OR = 0.40 (0.27, 0.59)], cucumber [27.7% vs. 11.9%, OR = 0.36 (0.27, 0.47)]; p-values < .001; Fig 1). Rates of classic tobacco (5.6% vs 4.2%, OR = 0.76 [0.48, 1.21], Virginia tobacco (5.7% vs. 4.3%, OR = 0.79 [0.48, 1.22]) and menthol (10.0% vs. 8.3%, OR = 0.85 [0.60, 1.21]) JUUL pod use did not change significantly (p-values > 0.05). The rate of mint JUUL pod use among current JUUL users increased from 2018 to 2019 (62.0% vs. 68.6%, OR = 1.40 [1.13, 1.73], p = 0.002).

With regard to the total number of flavors used, past 30-day JUUL users reported using fewer flavors in the past 30 days in 2019 compared to 2018 (2018: M = 2.09 [*SD* = 1.71]; 2019: M = 1.50 [*SD* = 1.33], t(1575) = 7.51, p < .001). The follow-up chi-square test indicated that rates of using no JUUL pod flavors increased from 2018 to 2019 (11.9% vs. 16.1%) as did the use of one JUUL pod flavor (32.8% vs. 47.0%). Rates of using two JUUL pod flavors were similar from 2018 to 2019 (24.2% vs. 21.0%). However, rates of using 3, 4, and 5 or more JUUL pod flavors in the past month decreased significantly from 2018 to 2019 (3 flavors: 15.3% vs. 9.4%; 4 flavors 7.3% vs. 3.2%; 5 or more flavors 8.6% vs. 3.2%). Crosstabs of the number of flavors used in the past month by frequency of JUUL use (separated by year) indicated that the pattern of results observed from the chi-square analysis reported above largely was consistent across different frequencies of JUUL use (see Table 1).

**Table 1:** Crosstabs of the total number of JUUL pod flavors used in the past month by frequency of JUUL use for 2018 and 2019.

	Once a week or less		2–3 times a week		4–6 times a week		Everyday	
# Flavors	2018	2019	2018	2019	2018	2019	2018	2019
0	20.7	**26.2**	4.7	**7.9**	7.2	**9.4**	4.1	**6.7**
1	45.4	**53.5**	33.6	**53.5**	22.5	**38.5**	17.2	**35.4**
2	22.3	13.8	30.5	23.7	29.7	28.1	21.3	28.7
3	7.9	**4.3**	16.4	**10.5**	20.7	**16.7**	23.8	**14**
4	2.1	**1.5**	7	**2.6**	11.7	**5.2**	13.5	**5.6**
≧ 5	1.6	**0.7**	7.8	**1.8**	8.1	**2.1**	20.1	**9.6**

Note. Bolded values indicate findings that are consistent with the direction of effects observed for the chi-square analysis examining changes in rates of total flavor use from 2018 to 2019.

## Discussion

The current study examined changes in Connecticut public high school students’ self-reported past-month use of JUUL devices, and, among past-month JUUL users, of JUUL pod flavors before and after voluntary sales restrictions were put into place by JUUL, Inc. in November 2018. Although it is possible that a variety of factors associated with the rapidly changing landscape of e-cigarette use or the self-report nature of the data impacted the use rates observed in the current study, we observed changes in rates of JUUL device use and the use of flavored JUUL pods that plausibly were related to the sales restrictions.

Among the full sample, the overall rate of past-month JUUL use decreased from 2018 to 2019, yet past-month JUUL use remained considerable at 26.5%. Given the study design, it was not possible to determine the extent to which the JUUL restrictions directly impacted overall rates of JUUL use, although it is plausible that the observed reduction in JUUL device use was linked to preferred flavor(s) becoming difficult to obtain.

Among current JUUL users, rates of past-month use of all four pod flavors that were restricted to online sales via JUUL’s website decreased. We also observed a significant decrease in the total number of flavors used in the past month, which largely was consistent across levels of JUUL use frequency. These findings suggest that JUUL’s voluntary sales restrictions on flavors that were popular among youth may have impacted the flavors that continued to be used by current JUUL users in a measurable way. However, the use of restricted JUUL pod flavors persisted, suggesting that the restrictions had a limited reach. Of particular concern, the post-restriction rate of mango JUUL pod use (36.9%) exceeded pre-restriction use rates for all other flavors except mint (range of unrestricted flavors 5.5–27.7%; mint = 62.0%). These findings raise the possibility that youth obtained restricted JUUL pods from sources such as JUUL’s website or friends/family; used pre-filled or refillable JUUL-compatible pods manufactured by a third party; or hacked and refilled single-use JUUL pods with flavors that mimicked the restricted flavors or with other substances like cannabis. Of note, we observed that some students who indicated past-month JUUL use reported that they did not use any flavored JUUL brand pods (2018: 11.9%; 2019: 16.1%), suggesting that youth may be using JUUL-compatible pods or hacking and refilling JUUL brand pods. However, these possibilities were not assessed explicitly in the current study and need further exploration.

As anticipated, rates of tobacco and menthol pod use remained low among current JUUL users and did not change significantly following the 2018 restrictions. These findings are consistent with research indicating that tobacco and menthol flavors do not have strong appeal to youth [7]. Importantly, following the restrictions mint JUUL pod use increased among past-month JUUL users. This finding suggests that some youth likely switched from using restricted flavors to mint. However, it was not possible to examine directly whether JUUL users switched from using restricted JUUL flavors to JUUL mint pods or switched from using JUUL to other devices because the data were cross-sectional and not matched over time. Thus, future research that employs a longitudinal, within-subjects design is needed to more clearly evaluate how restrictions on certain flavors impact the use of unrestricted flavors and/or other devices. Finally, the analytic sample was limited to Connecticut public high school students who reported current JUUL use, which may limit its generalizability. However, the two surveys included in the current study are part of a programmatic line of research that has been conducted for nearly a decade. Historically, rates observed in our data mirror national data closely [3]. Thus, the increased popularity of mint but not menthol JUUL pods among current JUUL users in our study suggests that the higher rates of mint/menthol flavor use observed in the 2019 NTYS [1] likely were driven by increased mint use.

## Conclusions

The current study focuses on the impact of JUUL Inc.’s 2018 voluntary flavor restrictions as one exemplar, but the findings suggest that broader flavor restrictions have the potential to impact e-liquid flavors used by young e-cigarette users. In the absence of a full ban on all non-tobacco flavors for all vaping products, adolescent e-cigarette users may switch to unrestricted flavors or obtain restricted flavors via other means. These findings are especially relevant to consider in light of the 2020 FDA prioritization of policy prohibiting the manufacture, distribution, or sale of pre-filled pods/cartridges in flavors other than menthol and tobacco pending premarket authorization [13]. While current restrictions may reduce youth use of cartridge-based devices or specific flavored cartridges/pods, they may lead to increased use of products like disposable vapes [14] or a resurgence in the popularity of unrestricted, bottled flavored e-liquids and devices like vape-pens and mods that are not subject to the current restrictions. An increase in the prevalence of using third-party refillable pods/cartridges or hacking single-use pods/cartridges to refill them with e-liquids that are not subject to current restrictions also may be observed. In addition, although mint flavored pods/cartridges are included in current restrictions, mint and menthol JUUL pods have been shown to be very similar to one another and to contain comparable menthol levels [15]. In the absence of clear regulatory guidance on what constitutes a permissible ‘menthol’ e-liquid flavor, it is possible that various ‘mint’ flavors could be repackaged and sold as ‘menthol’ under the existing policy. However, given the high rate of mint relative to menthol use observed among youth, additional research is needed to explain why this discrepancy exists. For example, the word “menthol” could be negatively associated with cigarette smoking while mint is not, making flavors labeled as “menthol” less appealing to youth than flavors labeled as “mint” irrespective of their underlying similarities. It also is possible that the discrepancy is due to menthol and mint flavors differing on other e-liquid constituents (e.g., sweeteners), suggesting that constituents which are featured more prominently in flavors traditionally labeled as “mint” should be banned or limited in products labeled as “menthol” to deter youth use. In sum, the results of the current study highlight the critical need for continued efforts to evaluate the impact of any decisions FDA makes regarding the permissibility of flavored e-liquids on youth vaping behavior.

## Supporting information

S1 Survey Question text and response options for the variables included in this study. (DOCX)

S1 Data Data file containing all the central study variables. (XLSX)
