# Supplementary material for: High school students’ use of JUUL pod flavors before and after JUUL implemented voluntary sales restrictions on certain flavors in 2018
Source: PLoS One. 2020 Dec 15;15(12):e0243368. doi: 10.1371/journal.pone.0243368 (PMC7737969; doi:10.1371/journal.pone.0243368)
Supplement: S1 Survey — (DOCX) [file pone.0243368.s001.docx]

Sex: At birth, what was your sex?

Female

Male

Age: How old are you?

13

14

15

16

17

18

19

Ethnicity: Are you Hispanic, Latino/a/x, or of Spanish origin?

No

Yes

Race: How do you describe your race? (select all that apply)

White

Black or African American

Asian or Asian American

American Indian or Alaska Native

Native Hawaiian or Pacific Islander

Middle Eastern

Other (please specify): ___________

Lifetime JUUL use: Have you ever tried a JUUL (picture of a JUUL included; rechargeable with a USB charger; has pods that insert into the device)?

No

Yes

*Asked only to participants who indicated lifetime JUUL use.

Past month JUUL use: Approximately how many days out of the past 30 days did you use a JUUL (picture of a JUUL included; rechargeable with a USB charger; has pods that insert into the device)?

0 (never) – 30 (everyday)

*Asked only to participants who indicated past-month JUUL use.

Past 30 day JUUL pod use: Which JUUL pod flavors did you use in the past 30 days? (Select all that apply)

Mango

Cool mint

Virginia tobacco

Cool cucumber

Classic menthol

Fruit medley

Crème brûlée

Classic tobacco

I have not used any of these flavors in the past 30 days (answer was exclusive)
